# Effects of Essential Oils and Polyunsaturated Fatty Acids on Canine Skin Equivalents: Skin Lipid Assessment and Morphological Evaluation

**DOI:** 10.1155/2013/231526

**Published:** 2013-11-06

**Authors:** S. Cerrato, L. Ramió-Lluch, D. Fondevila, D. Rodes, P. Brazis, A. Puigdemont

**Affiliations:** ^1^UNIVET S.L., Edificio Astrolabio, Avenue Cerdanyola 92, 08172 Sant Cugat del Vallés, Barcelona, Spain; ^2^Department of Medicine and Animal Surgery, Veterinary Faculty, Autonomous University of Barcelona, 08913 Cerdanyola del Vallès, Barcelona, Spain; ^3^MERIAL, Avenue Tony Garnier 29, 69007 Lyon, France; ^4^Department of Pharmacology, Therapeutics and Toxicology, Veterinary Faculty, Autonomous University of Barcelona, 08913 Cerdanyola del Vallès, Barcelona, Spain

## Abstract

A canine skin equivalent model has been validated for the assessment of a topical formulation effects. Skin equivalents were developed from freshly isolated cutaneous canine fibroblasts and keratinocytes, after enzymatic digestion of skin samples (*n* = 8) from different breeds. Fibroblasts were embedded into a collagen type I matrix, and keratinocytes were seeded onto its surface at air-liquid interface. Skin equivalents were supplemented with essential oils and polyunsaturated fatty acid formulation or with vehicle. Skin equivalents were histopathologically and ultrastructurally studied, and the three main lipid groups (free fatty acids, cholesterol, and ceramides) were analyzed. Results showed that the culture method developed resulted in significant improvements in cell retrieval and confluence. Treated samples presented a thicker epidermis with increased number of viable cell layers, a denser and compact *stratum corneum*, and a more continuous basal membrane. Regarding lipid profile, treated skin equivalents showed a significant increase in ceramide content (51.7 ± 1.3) when compared to untreated (41.6  ±  1.4) samples. Ultrastructural study evidenced a compact and well-organized *stratum corneum* in both treated and control skin equivalents. In conclusion, cell viability and ceramides increase, after lipid supplementation, are especially relevant for the treatment of skin barrier disruptions occurring in canine atopic dermatitis.

## 1. Introduction

In recent years, significant progress has been made to produce engineered substitutes of human and animal skin [1]. Human skin equivalents (SE) reconstructed from both epidermal and dermal compartments are currently employed for safety and toxicity studies in both cosmetics and pharmaceutical compounds [2]. 

In veterinary medicine, tissue culture technology has been mainly applied to the development of *in vitro* models of canine skin pathologies such as epidermolysis bullosa [3]. Afterwards, Serra et al. [4] developed an SE from healthy canine skin suggesting the use of that organotypic skin culture as an alternative to the *in vivo* investigations for skin research. Nevertheless, to our knowledge, there are no studies evaluating the suitability of canine SE models for pharmacological compounds testing. A canine SE would be a useful tool for test topical or systemic treatments in veterinary medicine contributing to cutaneous drug research while sparing experimental animals. In this way, it would be possible to study the effects not only in skin cells but also in skin morphology and functionality. 

Atopic dermatitis (AD) is the most common skin disease in veterinary medicine and, as other dermatoses, like irritant dermatitis, allergic contact dermatitis, and ichthyosis, it is characterized by an epidermal barrier defect [5–8]. Impaired skin allows an excessive penetration of allergens and microorganisms that may trigger the acute and chronic inflammatory response of AD [9–12].

Skin barrier defect could be caused by several phenomena such as a decrease of lipid matrix production from stratum corneum (SC) or alterations in the relationships between the three SC major lipids of the matrix (ceramides (CER), fatty acids (FA) and cholesterol (CHO)) [13–15]. Epidermal ultrastructural changes have been also related to AD as abnormal intercorneocyte junctions [12, 16] and abnormal cellular maturation and differentiation [17–19]. 

Topical administration or diet supplementation with free fatty acids (FFA) could stimulate the production of endogenous lipids contributing to the formation and improvement of epidermal barrier [10, 11, 20–22]. 

Essential oils and polyunsaturated fatty acids have demonstrated effective results not only in atopic dogs, showing reduced mean CADESI and pruritus scores [23, 24], but also in atopic cats [25].

The purpose of the present study was to assess the suitability of canine SE model as a tool to evaluate the influence of topical treatments on epidermis development. With this objective, essential oils and polyunsaturated fatty acids were used to investigate the changes induced on the lipid profile, epidermis development, and basal membrane structure of canine SE.

## 2. Material and Methods

### 2.1. Skin Equivalent Development

Cutaneous cells were isolated from fresh skin biopsies obtained from eight healthy dogs between 1–12 years old. Abdominal samples were taken from other purposes surgeries at the Veterinary Hospital of the Universitat Autònoma de Barcelona. During the present study, skin samples (*n* = 8), obtained from different dog breeds (Beagle, Great Dane, Weimaraner, West Highland White Terrier, and crossbreed), were processed.

Samples were washed in phosphate buffered saline, cut in small fragments (1 mm^3^), and digested with collagenase type I solution (2 mg/mL) in DMEM for 4–6 h at 37°C until dermis were totally digested. Collagenase supernatants was washed and centrifuged at 300 g for 5 min, and the obtained fibroblasts were grown in a humidified atmosphere at 37°C with 5% CO_2_ during 2 days. Medium was changed twice a week, and cells were used between passages 2 to 5.

After collagenase digestion, remaining epidermal fragments were washed and digested with a solution of 0.05% trypsin-0.02% EDTA during 30 min at 37°C, in order to obtain a high pure culture of keratinocytes. Trypsin supernatants were filtered twice with cell strainers (100 *μ*m and 40 *μ*m pore size, resp, BD Biosciences, Bedford, MA, USA) and centrifuged at 300 g for 5 min to recover keratinocytes. 

Primary keratinocytes (1 × 10^6^ cells/cm^2^) were plated in collagen coated flasks with mitomycin C-inactivated 3T3 cell feeder layer in a humidified atmosphere at 37°C with 5% CO_2_ for 1 week in DMEM/F12 (3 : 1) with 10% FCS (Gibco), 10^−6^ M hydrocortisone, 10^−6^ M isoproterenol, and 10^−7^ M insulin (Sigma). 

To obtain a three dimensional SE, 3 mL of rat tail type I collagen solution (1.5 mg/mL solution) containing 1.2 × 10^5^ fibroblasts were placed in a transwell chamber (Corning Tewksbury, MA, USA). The biomatrix was cultured for 5–7 days, and then 5 × 10^5^ keratinocytes were seeded onto its surface. After 24 hours, SEs were lifted at the air-liquid interface, and the medium was modified by including 1% of FCS, L-serine 0.1%, FFA (15 *μ*M linoleic acid, 7 *μ*M arachidonic acid, and 25 *μ*M palmitic acid), 50 *μ*g/mL ascorbic acid, 1 *μ*M DL-*α*-tocopherol-acetate, and 2.4 × 10^−5^ M BSA (all of them from Sigma). Skin equivalents were cultured for 48 h, and then the same medium but without FCS and 30 *μ*M linoleic acid was used. Skin equivalents were cultured, a total of 14 days at air-liquid interface, and medium was changed twice a week. 

### 2.2. Skin Equivalent Treatments

A total of 22 SEs were developed, 13 treated and 9 controls. Seven days after seeding the keratinocytes, SEs medium were supplemented with 10 *μ*L (1% in 9% ethanol aqueous solution/mL of culture medium) of essential oils and polyunsaturated fatty acids formulation, *Rosmarinus officinalis* leaf oil, *Lavandula hybrida* oil, *Eugenia caryophyllus* bud oil, *Melaleuca alternifolia* leaf oil, *Cinnamomum camphora* leaf oil, *Mentha piperita* oil, *Cedrus atlantica* bark oil, *Curcuma longa* root oil, *Origanum compactum* oil, *Gaultheria procumbens* leaf oil, *Cannabis sativa* seed oil, *Melia azadirachta* seed oil (*Dermoscent Essential 6, *Laboratoire de Dermo-Cosmétique Animale, France). Control SEs were developed in the identical condition but treated with vehicle. 

### 2.3. Histopathological Study

A total of 12 SEs were used to perform the histopathological studies: 7 treated and 5 controls. Skin equivalents and healthy canine skin as control were fixed in 10% formalin and embedded in paraffin wax. Sections (4 *μ*m) were cut and stained by routine methods with hematoxylin and eosin (H&E). Moreover, pancytokeratin (cytokeratins 5, 6, 8, and 17–19), collagen type IV, laminin 5, and vimentin were used to characterize the structure of epidermis, dermis, and basement membrane in SE models. 

After deparaffinization and hydration, samples were treated with 3% H_2_O_2_ (33%) to inhibit the endogenous peroxidase, and the nonspecific sites were blocked with 2% BSA in TBS for 1 h at RT before the addition of each primary antibody. The primary antibodies used, with the corresponding pretreatment for antigen retrieval and dilution conditions, were mouse anti-pancytokeratin (pretreatment of 0.01% trypsin for 20 min at RT, 1 : 25 dilution, Clone MNF116; Dako, Carpinteria, CA, USA), goat anti-collagen IV (pretreatment of protease type XIV for 8 min at 37°C, 1 : 100 dilution; Southern Biotechnology, Birmingham, AL, USA), rabbit anti-laminin 5 (pretreatment of 0.1% proteinase K for 8 min at 37°C, 1 : 800 dilution; Dako, Carpinteria, CA, USA), and mouse anti-vimentin (pretreatment of citrate buffer for 20 min at 98°C plus 30 min at RT, 1 : 50 dilution, Clone V9; Dako, Carpinteria, CA, USA). After incubation with the corresponding primary antibody (16 h at 4°C), samples were processed using a streptavidin-biotin complex. Secondary biotin-labelled antibodies used were goat anti-mouse IgG for pancytokeratin and vimentin, a rabbit anti-goat IgG for collagen IV, and a goat anti-rabbit IgG for laminin 5. All the secondary antibodies were used at a 1 : 200 dilution in TBS for 1 h at RT and were from Dako (Carpinteria, CA, USA). Diaminobenzidine (DAB) was used as the detection system and, finally, samples were counterstained with haematoxylin.

### 2.4. Functional Study: Lipid Quantification

Two weeks after starting the treatment, SEs were removed and the lipid profile was assessed. A total of 10 SEs were used, 6 SEs supplemented with the essential oils and polyunsaturated fatty acids formulation and 4 controls. Treated SEs were harvested, and material from the epidermis and dermis compartment was extracted according to Bligh and Dyer method [26]. Briefly, lipids were extracted from the epidermis at RT by stirring with different mixtures of chloroform/methanol 2 : 1, 1 : 1, and 1 : 2 for 2 hours each. Samples were dried with nitrogen and dissolved again in chloroform/methanol 2 : 1. The quantitative analyses of lipids from obtained extracts were performed by thin layer chromatography coupled to an automated ionization detector (Iatroscan MK-5 analyzer, Iatron, Tokyo, Japan). Samples were applied on Silica gel S-III Chromarods using a SES (Nieder-Olm, Germany) 3202/15-01 sample spotter. Before performing a total scan, the rods were developed to a distance of 2.5 cm with chloroform/methanol/water (57 : 12 : 0.6), to 8 cm with hexane/diethyl ether/formic acid (50 : 20 : 0.3), and finally to 10 cm with hexane/benzene (35 : 35) [27].

The total lipids were separated in the following 3 fractions: CHOL, FFA, and CER. For each sample the analyses were performed in duplicate. 

### 2.5. Ultrastructural Study: Electron Microscopy

Six canine SEs were processed for electron microscopy to evaluate the ultrastructure in control (*n* = 3) and treated samples (*n* = 3). Samples were immersed in a fixative solution of 2% (w/v) paraformaldehyde (EM grade, Merck, Darmstadt, Germany) and 2.5% (v/v) glutaraldehyde (EM grade, TAAB Laboratories, Berkshire, UK) in phosphate buffer (PBS; 0.1 M, pH 7.4; Sigma Aldrich, Steinheim, Germany) for 2 h. 

Following fixation, the samples were rinsed four times with 100 mM PBS. Samples were then postfixed in 1% (w/v) osmium tetroxide (TAAB) containing 0.8% (w/v) potassium hexacyanoferrate (III) (Sigma, Madrid, Spain) for 2 h and washed with deionized water four times and sequential dehydration in acetone. All procedures were performed at 4°C. Samples were dehydrated through a graded acetone series, embedded in Eponate 12 resin (Ted Pella Inc., Redding, California), and polymerized for 48 h at 60°C. 

Semithin sections (1 mm thick) were obtained with a Leica ultracut UCT microtome (Leica Microsystems GmbH, Wetzlar, Germany), stained with 1% (w/v) aqueous toluidine blue solution, and examined with a light microscope to identify areas for the following steps.

Ultrathin sections (70 nm in thickness) were cut with a diamond knife (45°, Diatome, Biel, Switzerland), mounted in copper grids (200 mesh), and contrasted with conventional uranyl acetate (30 min) and Reynolds lead citrate (5 min) solutions. Sections were observed with a Jeol 1400 transmission electron microscope (Jeol Ltd, Tokyo, Japan) equipped with a Gatan Ultrascan ES1000 CCD Camera (Gatan, Inc. CA, USA).

### 2.6. Statistical Study

Statistical significance of the results was determined using the two tailed Student's *t*-test for unpaired data (*P* < 0.05 and *P* < 0.01).

## 3. Results 

### 3.1. Skin Equivalent Obtaining

Keratinocyte isolation after collagenase digestion showed higher recovery rates than dispase digestion obtained in our lab previously (unpublished results). Hence, with collagenase digestion, a mean of 19.3 × 10^6^ cells/gr of skin was obtained whilst dispase digestion mean recovery was 4.8 × 10^6^ cells/gr of skin. In the same way, collagen coated flasks and coculture with mitomycin C- inactivated 3T3 feeder layer improved cell growth and full confluence achievement were obtained in a shorter period (7–9 days versus 14-15 days). This new technique allowed the establishment of several pure keratinocytes lines, with no fibroblast contamination and high viability rates (96–98%). 

### 3.2. Histopathological Study

**Figure 1:**
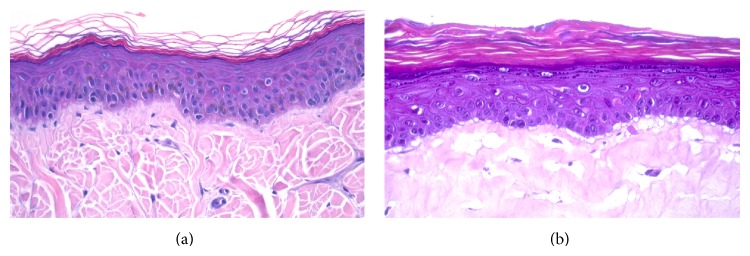
Histological sections of canine healthy skin (a) and canine skin equivalent (b) (400x).

The structure of the SEs, dermis and epidermis, was analysed after hematoxylin-eosin staining. Canine skin equivalents presented a similar morphological structure than healthy canine skin (Figure 1) as it was demonstrated in a previous study performed in our laboratory [4]. 

**Figure 2:**
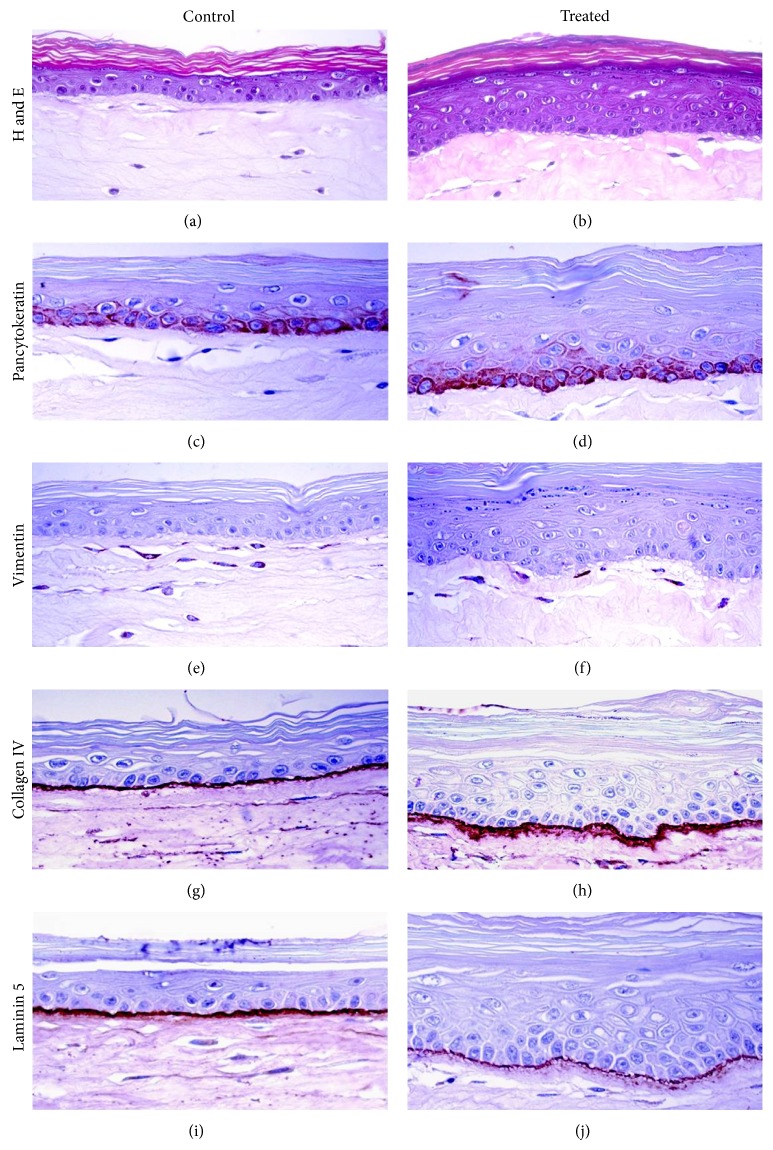
Morphological analysis of control and treated skin equivalents (400x).

Histological analysis showed an epithelial architecture resembling native skin with formation of a mature dermis and multilayered and differentiated epidermis, including the basal, spinous, and granular layers and a flattened stratum corneum in the overall SEs studied. As shown in Figures 2(a) and 2(b), differentiation and maturation of the dermis-equivalent (collagen gel biomatrix) and the epidermis-equivalent (keratinocytes layers), as well as the dermoepidermal junction, were correctly developed in both control and treated SEs. 

When essential oils and polyunsaturated fatty acids formulation were included in the medium, several differences were observed. In treated SEs, a more continuous basal membrane and an increase in number of keratinocyte layers was evidenced. Moreover, the SC was more dense and compact in treated SEs. 

The characterization of keratinocytes in basal and suprabasal layers of the SEs was performed by the immunodetection with pancytokeratin. The expression of specific epitopes of keratins was recognized in the cellular membrane by pancytokeratin antibody, demonstrating the correct maturation of the epidermis in SEs (Figures 2(c) and 2(d)). Moreover, the lack of reactivity of pancytokeratin antibody in the collagen gel bio-matrix agreed with the absence of cytokeratins expression in native dermis. 

The identification of fibroblasts of the SEs was performed by immunodetection of vimentin, a specific marker of mesenchymal cells, like fibroblasts. Vimentin-immunoreactivity was detected in the SE dermis in both, non-treated and treated samples, showing a properly structured, active, and mature dermis (Figures 2(e) and 2(f)). The lack of reactivity of vimentin antibody in keratinocyte layers agreed with nonexpression of vimentin in native epidermis. 

The development of a continuous basal membrane in dermoepidermal junction of SEs is observed in Figures 2(g) and 2(j). The presence and localization of the major basal membrane components, such as collagen type IV and laminin 5, were detected by immunohistochemistry. As shown in both control and treated SEs, a well defined membrane was observed; however, in treated samples the membrane thickness was higher than in non-treated skins especially for collagen type IV (Figure 2(h)).

### 3.3. Lipid Quantification

Samples obtained from canine SEs were analysed for lipid quantification. Lipids were separated in the following fractions: CHOL, FFA, and CER.

**Figure 3:**
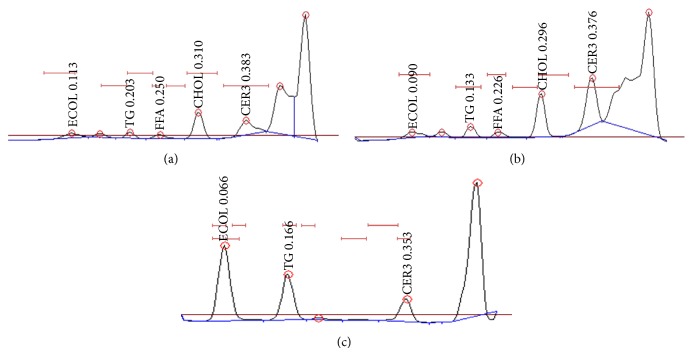
Chromatography study of the different lipid fractions of non-treated skin equivalents (a), essential oils and polyunsaturated fatty acids treated skin equivalents (b), and standard lipid profile (c).

In Figure 3 the chromatographic lipid profile of both treated and non-treated SEs can be observed. 

Table 1 shows the percentage of different lipid fractions obtained from the lipid extract of canine SEs with and without treatment. As observed, when canine SEs were treated with the essential oils and polyunsaturated fatty acids formulation, the ceramide percentage was significantly higher (51.7 ± 1.3) compared to that in control samples (41.6 ± 1.4). Moreover, a significant decrease in FFA was observed in treated skin samples.

### 3.4. Ultrastructural Study

**Figure 4:**
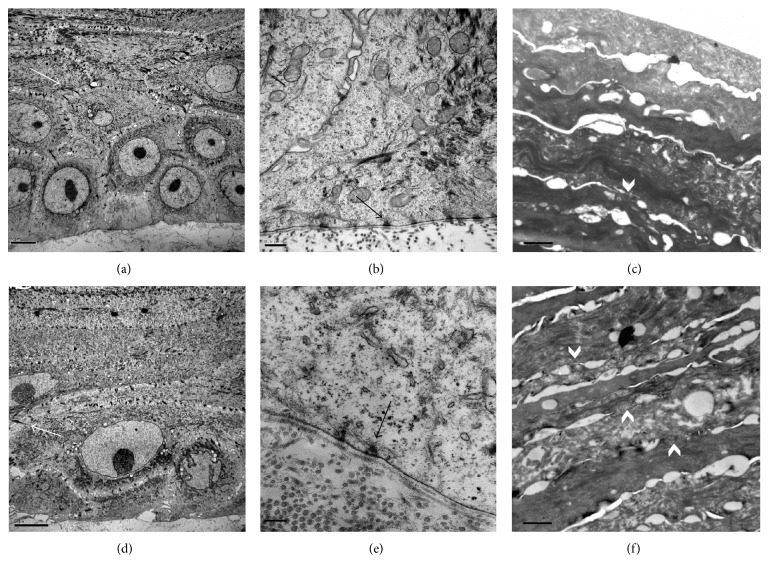
Ultrastructural images of non-treated (a, b, c) and treated skin equivalent with essential oils and polyunsaturated fatty acids (d, e and f). Desmosomes between adjacent corneocytes (a, c) (white arrows), hemidesmosomes (b, e) (black arrows), and stratum corneum with vertical cohesion between corneocytes layers (white arrow head) (c, f) were observed in both samples. Scale bars: 5 *μ*m (a and d), 0.5 *μ*m (b), 1 *μ*m (c and f), and 0.2 *μ*m (e).

Ultrastructural morphology of four SEs was examined by transmission electron microscopy. Untreated and treated SEs presented thick and well developed desmosomes between corneocytes in the different strata (Figures 4(a) and 4(d)). In dermal-epidermal junction, a continuous basal membrane with both densa and lucida laminae was observed. Moreover, hemidesmosomes between basal corneocytes and basal membrane were observed in both treated and non-treated samples (Figures 4(b) and 4(e)). 

Stratum corneum was also well-organized, compact, and dense with almost entirely fulfilment of intercorneocyte spaces (Figures 4(c) and 4(f)) in both, treated and non-treated samples. Even though no morphological differences were observed between samples, during TEM sample processing, samples treated with essential oils presented a more resistant SC bindings than control samples that were often separated from the upper layers of epidermis, while in treated samples SC was always attached to epidermis. 

## 4. Discussion 

The results shown in the present study demonstrated the suitability of canine SE model to investigate the effects of topical essential oils and polyunsaturated fatty acids, in canine epidermis. In the present study, the structure and lipid composition were also analysed. A significant increase in CER concentration of SE lipids was observed after the treatment.

Skin equivalents have been developed and investigated over the past years for their applications in human medicine for skin biology research [28, 29], cutaneous irritation, and toxicity testing [30, 31], and as experimental models for permeability and cutaneous absorption of different agents and formulations [18, 32]. On the contrary, in veterinary medicine, little information is published regarding SEs. Canine organotypic skin cultures have been developed for skin dysfunctions studies, such as epidermolysis bullosa or k10 lack-keratinisation defect [3, 33]. These canine SEs were designed as disease research-models. The characterization of healthy canine SE model was not performed until 2007. Serra et al. developed a canine SE from healthy skin suggesting the use of that organotypic skin culture as an alternative to *in vivo* investigations for skin research. In the present study, several changes have been incorporated in the model described by Serra et al. [4] in order to optimize the technique. 

First of all, regarding the obtaining method, the isolation of keratinocytes after dermal collagenase separation showed in the present study resulted in increased recovery rates when compared with the classical dispase digestion, probably due to higher hair follicle stem cell retrieval. This hypothesis is based on the fact that epidermis-dermis separation with dispase requires epidermis sheet to be mechanically peeled from the dermis, and probably a high percentage of hair follicles remains in the dermis portion. Contrarily, by using collagenase in the process, dermis is first digested, and then the resultant fraction is composed by the total epidermis, including hair follicles with proper stem cells. Another improvement achieved through the present method was the coculture of keratinocytes with mitomycin C-inactivated 3T3 feeder layer in collagen coated flasks that showed a shorter confluence period in primary cultures in comparison with noncoated flasks used by Serra et al. [4]. Therefore, in this study, keratinocytes isolation and culture has been optimized regarding the primitive model allowing easier and faster SE obtaining. 

This improved SE has been used to test the effects of a lipidic formulation for the improvement of canine skin condition. With this purpose, the lipidic compound was added to the SE growth medium in order to determine its effects in basal layers development, maturation, and differentiation. Protein expression and healthy skin ultrastructure were not altered by lipid supplementation in growth medium, but an increase in the number of epidermal layers in treated samples was observed, probably due to an increase in the viability. This hypothesis agrees with previous studies evidencing fatty acid supplementation in culture media improves cell growth and promotes epithelial morphogenesis in SEs [28, 34].

As described previously, modifications in culture media can lead to changes not only in the differentiation process but also in lipid profile of the SE [28, 32, 35, 36]. In the present study, three lipidic groups were analyzed (CHOL, FFA, and CER). Essential oils and polyunsaturated fatty acids supplementation induced an increase in the CER proportion of epidermal lipids. Fatty acid supplementation has already been described to promote CER synthesis since CER are generally composed of a sphingoid base and fatty acids [28, 37, 38]. This point is important since CER level has been demonstrated to be decreased in diseases related to impaired skin barrier such as AD [14, 39–41]. Moreover, essential fatty acids can reduce allergic inflammation response through the modulation of prostaglandin and leukotriene production [42, 43] and the inhibition of cellular activation and cytokine secretion [44], as well as the alteration of the composition and function of the epidermal lipid barrier [11]. Therefore, the results of the present study agree with previous studies where topical and dietary administration of essential oils and polyunsaturated fatty acids have been closely related to the improvement of the lipidic matrix of canine atopic skin and its symptomatology [11, 22, 23, 45, 46]. 

Although FFA were present in the topical formula used for the treatment, their levels decreased in treated SEs samples. This result suggests FFA incorporation to the SE lipids as the substrate for the CER synthesis or by binding to epidermal proteins as it has been described previously [18, 47]. In fact, FFA reduction observed in treated samples was not reflected in an alteration of SC structure since electron micrographs showed compact and dense lipid lamella in treated as in control SEs. 

In the present study, the effects of essential oils and polyunsaturated fatty acids were studied in a healthy SE model. So, the effects of lipidic supplementation would be more evident and the clinical benefit would be more measurable if AD animals with skin barrier dysfunction and epidermal lipid disorders were used. However, canine SE is a useful model to evaluate and to extrapolate the activity of exogenous compounds such as lipidic formulations on epidermal lipid matrix and the potential contribution of such active molecules, avoiding the use of live dogs.

In this study we described for the first time the suitability of canine SE for testing topical treatments contributing to cutaneous drug research while sparing experimental animals. With this model it is possible to test the effects not only in skin cells but also in skin morphology, functionality, and changes in extracellular lipid composition. 

In conclusion, the purposes of this study have been achieved since the SE has showed to be an appropriate model for the study of dog skin. Moreover, this canine SE model enables the study of the effects of compounds such as essential oils and polyunsaturated fatty acids, showing an increase in CER content of intercellular lipid domain of SC, which becomes particularly relevant in the treatment of atopic dermatitis and other lipid barrier dysfunctions.

## Figures and Tables

**Table 1:** Percentage of different lipid fractions obtained from the skin equivalents in control (*n* = 4) and treated samples (*n* = 6).

Lipid fractions (%) of skin equivalents		
Lipid profile	Control (MEAN ± SEM)	Treated (MEAN ± SEM)
FFA	21.6 ± 1.1	15.9 ± 1.3 ∗∗
CHOL	36.7 ± 1.5	32.4 ± 1.7
CER	41.6 ± 1.4	51.7 ± 1.3 ∗∗

^**^ *P* < 0.01.
